# Treatment with non-automated insulin pumps or multiple daily injections during pregnancy and post-delivery in women with type 1 diabetes: A secondary analysis of the CopenFast trial

**DOI:** 10.1007/s00592-025-02560-w

**Published:** 2025-08-04

**Authors:** Katrine Christiansen, Sidse K. Nørgaard, Kirsten Nørgaard, Tine D. Clausen, Peter Damm, Elisabeth R. Mathiesen, Lene Ringholm

**Affiliations:** 1 Department of Nephrology and Endocrinology, Rigshospitalet, Copenhagen, Denmark; 2 Department of Clinical Medicine, Faculty of Health and Medical Sciences, University of Copenhagen, Copenhagen, Denmark; 3 Center for Pregnant Women with Diabetes, Rigshospitalet, University of Copenhagen, Blegdamsvej 9, Copenhagen, 2100 Denmark; 4 Novo Nordisk, Søborg, Denmark; 5 Steno Diabetes Center Copenhagen, Herlev, Denmark; 6 Department of Gynaecology, Fertility, and Obstetrics, Rigshospitalet, Copenhagen, Denmark

**Keywords:** Pregnancy, Type 1 diabetes, Non-automated insulin pumps, Continuous glucose monitoring, Preterm delivery, Caesarean section

## Abstract

**Aim:**

To evaluate pregnancy and post-delivery outcomes with non-automated insulin pumps or multiple daily injections (MDI) in women with type 1 diabetes (T1D).

**Methods:**

A preplanned secondary analysis of the CopenFast trial including women with T1D using continuous glucose monitoring (CGM), primarily intermittently scanned CGM, routinely. Pregnancy and post-delivery outcomes were compared between insulin pump users and MDI users. Insulin pump settings during pregnancy and post-delivery were compared to pre-pregnancy.

**Results:**

Of 141 women, 39 used non-automated insulin pumps and 102 used MDI. Median diabetes duration was 17 (IQR 12–20) vs. 14 (8–21) years (*P* = 0.12). HbA_1c_ was 48 (44–53) mmol/mol vs. 47 (42–53) at 9 weeks (*P* = 0.65) and 43 (40–46) vs. 43 (39–46) at 35 weeks (*P* = 0.53). Mean sensor glucose decreased from ~ 7.0 mmol/l at 9 weeks to 6.3 mmol/l at 33 weeks in both groups. Preterm delivery (< 37 weeks) was more common with insulin pumps (25.9% vs. 16.7%, *P* = 0.01), as was caesarean section (59% vs. 40%, *P* = 0.04), which was independent of diabetes duration, age and microvascular complications. At 1 and 3 months post-delivery, maternal and infant outcomes, including breastfeeding, were similar between groups. In insulin pump users, basal insulin rates were ~ 37% higher, and carbohydrate-to-insulin ratios ~ 61% lower at 33 weeks while basal insulin rates were ~ 20% lower, and carbohydrate-to-insulin ratios were similar 3 months post-delivery, compared to pre-pregnancy.

**Conclusion:**

Despite routine use of CGM and similar glycaemic control, non-automated insulin pump users did not achieve improved pregnancy outcomes compared to MDI users.

## Introduction

Women with type 1 diabetes are at increased risk of adverse pregnancy outcomes, such as preterm delivery, fetal overgrowth and neonatal morbidity compared to the background population [1–6]. Appropriate insulin treatment and diet are important to obtain and maintain optimal glycaemic control before and during pregnancy while preventing maternal hypoglycaemia [7].

In non-pregnant persons with type 1 diabetes, insulin pump treatment can lead to improved glycaemic control with a reduced risk of severe hypoglycaemia compared to multiple daily injections (MDI) [8, 9]. This is particularly distinct in women [8]. In Denmark, women with type 1 diabetes generally continue their usual insulin pumps or MDI during and after pregnancy, while initiation of insulin pumps during pregnancy is rare [10–13].

Throughout pregnancy, insulin requirements change often with gradual increase from ~ 16 weeks, necessitating regular adjustments of basal rates and insulin-to-carbohydrate ratios [14, 15, 16]. However, only one small study has addressed adjustments of the specific insulin pump settings during pregnancy in non-automated insulin pumps, where basal insulin is administered continuously at fixed rates and where bolus insulin is administered manually [10]. Immediately after delivery, insulin requirements drop to 50–70% of pre-pregnancy insulin dose and remain ~ 20% lower in the following months [11, 17, 18]. Insulin pumps include several complex features that could, theoretically, accommodate these needs for frequent insulin dosing adjustments during pregnancy [10], but, surprisingly, insulin pumps without combined use of continuous glucose monitoring (CGM) are not superior to MDI during pregnancy for optimising glycaemic control or pregnancy outcomes [12, 19, 20]. In contrast, the use of CGM has favourable effects on pregnancy outcomes compared to self-monitoring of blood glucose [21]. Women with type 1 diabetes are offered CGM when pregnant or planning pregnancy in several European countries, including Denmark [22].

We hypothesised that the combined use of non-automated insulin pumps and CGM during pregnancy in women with type 1 diabetes would lead to improved pregnancy outcomes, compared to women using MDI and CGM.

The aim of this study was to evaluate pregnancy and post-delivery outcomes with non-automated insulin pumps or MDI in women with type 1 diabetes with focus on glycaemic control during pregnancy and post-delivery, as well as pregnancy, neonatal and infant outcomes.

## Methods

### Trial design and patients

This was a preplanned secondary analysis based on prospectively collected data from the CopenFast trial, an open-label, single-centre, randomised controlled trial (RCT) evaluating the effect of faster-acting insulin aspart versus insulin aspart on pregnancy outcomes in women with type 1 or type 2 diabetes, followed from early pregnancy until 3 months post-delivery. Data were collected from November 2019 to March 2023 [23]. The women were stratified according to diabetes type (type 1 or type 2) and insulin treatment modality (insulin pump or MDI) and continued their usual insulin pumps or MDI and usual CGM during the trial [23, 24].

In the original trial, 146 of 203 (72%) women, who delivered a liveborn infant, had type 1 diabetes [23]. Five women did not use CGM during pregnancy and were therefore excluded, leaving 141 women with type 1 diabetes for this analysis, i.e. 39 on insulin pumps and 102 on MDI.

All insulin pumps were non-automated: Omnipod Eros 62% (*n* = 24) or Medtronic 38% (*n* = 15) (Minimed 640G (*n* = 7), Minimed 670G (*n* = 4), Minimed 515 (*n* = 2), Veo 554 (*n* = 2)). Among insulin pump users, 67% (*n* = 26) used intermittently scanned CGM (isCGM) and 33% (*n* = 13) used real-time CGM (rtCGM). In the MDI group, 89% (*n* = 91) used isCGM and 11% (*n* = 11) used rtCGM  .

Before randomisation, all women used insulin aspart as mealtime insulin, except one woman on insulin pump using faster-acting insulin aspart, and six women on MDI using faster-acting insulin aspart (*n* = 4), glulisine (*n* = 1) or lispro (*n* = 1).

### Routine diabetes and pregnancy care

All women attended the routine pregnancy care programme offered at our centre, which included consultations approximately every 2 weeks with a diabetes specialist until delivery, and as part of the trial, at 1 and 3 months post-delivery. Insulin pump users were mainly consulted by diabetologists E.R.M and/or L.R.

The women were recommended to aim for blood glucose levels 4.0-5.5 mmol/l preprandially, 4.0–7.0 mmol/l postprandially and 5.0–7.0 mmol/l before bedtime. Regardless of the use of insulin pumps or MDI adjustments of basal insulin doses and mealtime insulin doses were made at the clinician’s discretion at the routine visits. All women were also advised to adjust these insulin doses every 3–5 days between visits, when necessary. Targets for CGM metrics were mean sensor glucose 5.0–6.0 mmol/l, time in range in pregnancy (TIRp) (3.5–7.8 mmol/l (in isCGM users: 3.9–7.8 mmol/l as provided in the average glucose profile report)) > 70%, time above range in pregnancy (TARp) (> 7.8 mmol/l) < 25%, and time below range in pregnancy (TBRp) (< 3.5 mmol/l) < 4%. HbA_1c_ targets were < 48 mmol/mol (6.5%) before 20 weeks and < 38 mmol/mol (5.6%) thereafter [23– 25].

All women were offered dietary guidance [22–24]. The advice on gestational weight gain was based on the recommendations from National Academy of Medicine (NAM) [26].

At 33 weeks, a diabetologist evaluated and documented each woman’s target glucose level (usually 4.0–7.0 mmol/l) and estimated her insulin dose during and immediately after delivery based on the clinician’s discretion and documented in a delivery plan, which took insulin treatment modality and mode of delivery into account [27].

### Data collection and definitions

Baseline data were registered at randomisation at 9 (range 9–11) weeks. Trial visits took place with the routine obstetric consultations at 21, 33, and 35 weeks where the following data were recorded: gestational age, total daily insulin doses along with the percentage distributed as basal insulin, HbA_1c_, weight, blood pressure, proteinuria and events of mild hypoglycaemia (manageable by the woman) and severe hypoglycaemia (requiring help from others) [23, 24, 28, 29].

For isCGM users, mean sensor glucose, TIRp, TARp and TBRp at randomisation, 21 and 33 weeks were available from the patient records.

The following insulin pump settings were collected pre-pregnancy, at randomisation, 21 weeks, 33 weeks, 35 weeks, immediately post-delivery, at 1 month and 3 months post-delivery: Basal insulin rates at 2 a.m., 5 a.m., 11 a.m. and 5 p.m., carbohydrate-to-insulin ratio and insulin sensitivity factor, both at 3 a.m., 8 a.m., 12 noon and 6 p.m., target plasma glucose, insulin action time and the daily amount of carbohydrate entered in the insulin pump [10, 11].

Gestational weight gain (difference between last weight measured before delivery and self-reported pre-pregnancy weight [30]) was categorised as excessive if exceeding the recommendations from NAM: ≥16.0 kg if pre-pregnancy BMI < 25 kg/m^2^, ≥ 11.5 kg if pre-pregnancy BMI 25–29.9 kg/m^2^ and ≥ 9.0 kg if pre-pregnancy BMI ≥ 30 kg/m^2^ [31].

Gestational age at delivery, birthweight, and offspring sex were documented and used to calculate the birthweight standard deviation (SD) scores, adjusted for gestational age and infant sex. The calculations were based on growth curves generally used in Scandinavia [32] to compare the infants’ weights to the background population.

Large (LGA) and small (SGA) for gestational age were defined as birthweight ≥ 90th percentile and ≤ 10th percentile, respectively [32].

Preeclampsia was defined as hypertension developed after 20 weeks with coexistence of proteinuria or symptoms from other organs [33].

Early preterm delivery was defined as delivery < 34 completed weeks, and preterm delivery was defined as delivery < 37 completed weeks.

At 1 and 3 months post-delivery, HbA_1c_, mild and severe hypoglycaemia, breastfeeding, infant hospitalisation, infant weight and length were recorded. Infant weight SD score at 1 and 3 months and maternal weight retention (> 5.0 kg compared with pre-pregnancy weight) at 3 months were calculated [23, 24].

### Statistical analysis

All data were given as mean (SD) or median (interquartile range (IQR)) for continuous variables and as numbers (percentage) for categorical variables.

To analyse categorical variables either Χ^2^-test or Fisher’s exact test were used to compare the outcomes, depending on the sample size. A two-sample t-test was used to analyse continuous variables if the data were normally distributed, otherwise a Mann-Whitney U test was used. In explorative analyses, insulin pump settings at the four pre-set time points, respectively, at randomisation, 21 weeks, 33 weeks, immediately post-delivery and at 3 months post-delivery were compared to pre-pregnancy by paired t-tests when analysing normally distributed continuous variables, otherwise Wilcoxon signed-rank tests were used.

A mean value of the insulin pump settings across the four pre-set time points was calculated to support a simpler comparison of insulin pump settings pre-pregnancy and across trial visits.

Univariate logistic regression analysis was conducted with preterm delivery (yes/no) and caesarean section (yes/no), respectively, as dependent variables and insulin pump (yes/no), diabetes duration (years), age (years), presence of microvascular complications (yes/no) at baseline as independent variables. To identify independent risk factors for preterm delivery and caesarean section, respectively, multivariate logistic regression analyses were applied including independent variables with P values < 0.1 in the univariate analyses. Results were given as odds ratio (OR) and 95% confidence interval (CI). Correction for multiple testing was not performed.

A two-sided P value < 0.05 was considered statistically significant.

All analyses and figures were performed using R version 4.4.1 (R Core Team, 2024, R Foundation for Statistical Computing, Vienna, Austria).

### Ethics

All women provided written consent prior to participation, including the partners’ regarding infants data collection. The trial protocol was approved by The Danish Medicines Agency (2018-004680-31) and the Regional Ethics Committee (H-19029966) and registered with ClinicalTrials.gov, NCT03770767 [23, 24].

## Results

The insulin pump users were significantly younger and diagnosed with diabetes at a younger age compared to MDI users. All other baseline characteristics including microvascular complications were similar between the groups (Table 1). The proportion of women randomised to faster-acting insulin aspart or insulin aspart was similar between the groups.

**Table 1:** Baseline characteristics in 141 women with type 1 diabetes using non-automated insulin pumps or multiple daily injections during pregnancy

	Insulin pumps *n* = 39	Multiple daily injections *n* = 102	*P* value
Age, years	28 (27–33)	32 (28–35)	0.02
Age at diabetes diagnosis, years	13 (10–17)	17 (11–26)	0.01
Duration of diabetes, years	17 (12–20)	14 (8–21)	0.12
Pre-pregnancy HbA_1c_, mmol/mol	53 (49–58)	52 (47–58)	0.44
Pre-pregnancy HbA_1c_, %	7.0 (6.6–7.5)	6.9 (6.5–7.5)	0.44
Baseline HbA_1c_, mmol/mol	48 (44–53)	47 (42–53)	0.65
Baseline HbA_1c_, %	6.5 (6.2–7.0)	6.5 (6.0–7.0)	0.65
Nordic origin	34 (87.2)	93 (91.2)	0.53
Gestational age, days	65 (60–72)	66.5 (62–77)	0.36
Nulliparous	24 (61.5)	48 (47.1)	0.12
Pre-pregnancy body mass index, kg/m^2^	25.1 (23.6–27.8)	24.8 (22.4–28.1)	0.40
Mealtime insulin after randomisation			
- *Faster acting insulin aspart*	19 (48.7)	52 (51.0)	0.81
- *Insulin aspart*	20 (51.3)	50 (49.0)	
Systolic blood pressure, mmHg	116.0 (± 9.4)	115 (± 11.8)	0.57
Diastolic blood pressure, mmHg	75.6 (± 7.6)	74.1 (± 7.2)	0.28
Diabetic retinopathy^a^	15 (45.5)	31 (34.8)	0.28
Proteinuria			
- Microalbuminuria, albumin/creatinine ratio 30–299 mg/g	2 (5.1)	3 (2.9)^c^	0.62
- Macroalbuminuria, albumin/creatinine ratio ≥ 300 mg/g	1 (2.6)	1 (1.0)	0.48
Number of mild hypoglycaemic events the previous week, prior to randomisation	7 (3–10)	5 (2–7.5)	0.30
Number of participants with severe hypoglycaemia, *the year preceding pregnancy*^b^	1 (2.6)	2 (2.1)	1.0
Number of participants with severe hypoglycaemia, *in early pregnancy*,* prior to randomisation*	0 (0.0)	3 (3.0)	0.56
Routine use of continuous glucose monitoring			
Intermittently scanned continuous glucose monitoring, total	26 (66.7)	91 (89.2)	0.001
- *Libre 1*	*24*	*85 *	
- *Libre 2*	*2*	*6*	
Real-time continuous glucose monitoring, total	13 (33.3)	11 (10.8)	
- *Dexcom*	*6*	*11*	
- *Guardian*	*6*	*0*	
- *Enlite*	*1*	*0*	
Folic acid intake	38 (97.4)	98 (98.9)	1.0

Data are presented as mean (standard deviation), median (interquartile range) or n (%). Data were available for > 97% of the women except: ^a^84.6% and 87.3%, ^b^95.1% in the multiple daily injection group. ^c^Two women with microalbuminuria did not have diabetic retinopathy, data on diabetic retinopathy was not available in one woman with microalbuminuria

**Fig. 1:**
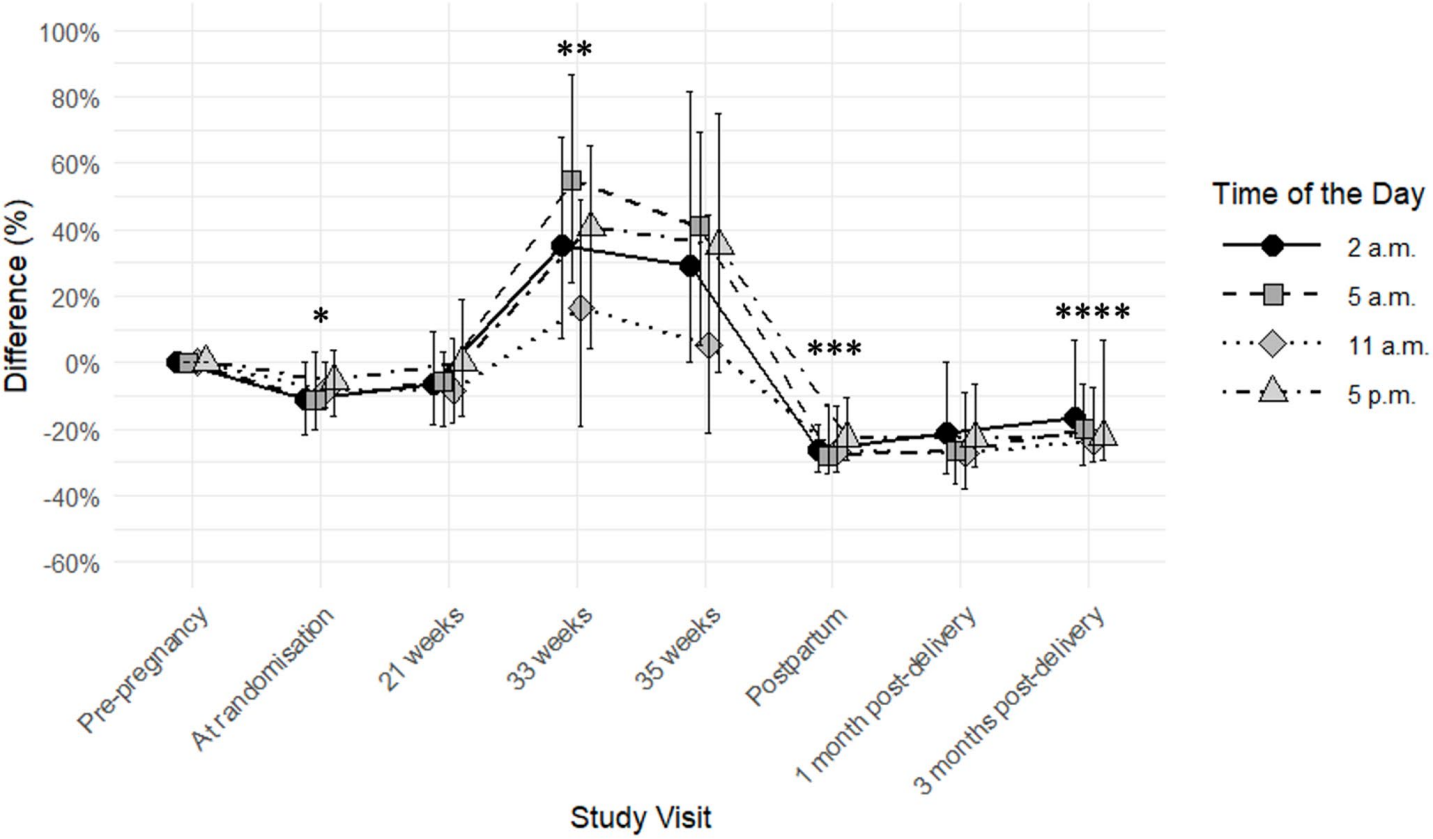
Difference in median [interquartile range] basal insulin rate (%), when compared to pre-pregnancy levels in 39 women with type 1 diabetes using non-automated insulin pumps. P values calculated by comparing to pre-pregnancy levels. *P ≤ 0.006, except at 5 p.m. which was *P* = 0.2. **all *P* ≤ 0.02. ***all *P* < 0.0001. ****all *P* ≤ 0.02

Compared to pre-pregnancy, the median basal insulin rates (Fig. 1) were ~ 9% lower at randomisation (all *P* ≤ 0.006, except at 5 p.m. where *P* = 0.2) and ~ 37% higher at 33 weeks (all *P*≤0.02). Immediately post-delivery, the basal insulin rates were ~ 26% lower than pre-pregnancy as estimated and documented in the delivery plan. At 3 months post-delivery, the basal insulin rates were ~ 20% lower compared to pre-pregnancy (all *P*≤0.02).

**Fig. 2:**
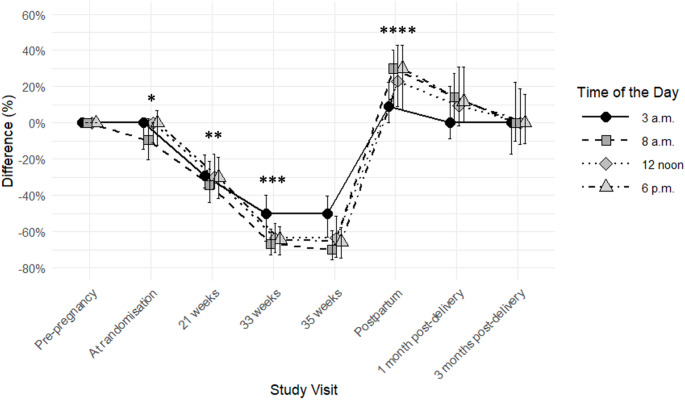
Difference in median [interquartile range] carbohydrate-to-insulin ratio (%), when compared to pre-pregnancy levels in 39 women with type 1 diabetes using non-automated insulin pumps. P values calculated by comparing to pre-pregnancy levels. *P ≤ 0.03, except at 12 noon and 6 p.m. which was 0.5 and 0.4, respectively. **all *P* < 0.0001. ***all *P* < 0.0001. ****all *P* ≤ 0.004

The median carbohydrate-to-insulin ratios at pre-pregnancy and randomisation were overall similar (Fig. 2). Compared to pre-pregnancy, the carbohydrate-to-insulin ratios were ~ 31% and ~ 61% lower at 21 weeks and 33 weeks, respectively (all *P* < 0.0001). Immediately post-delivery, the carbohydrate-to-insulin ratio was ~ 23% higher than pre-pregnancy as estimated and documented in the delivery plan. At 3 months post-delivery, the carbohydrate-to-insulin ratios were back at pre-pregnancy levels.

The changes in insulin sensitivity factor followed the same pattern as for carbohydrate-to-insulin ratios.

Target plasma glucose in the insulin pumps was 5.6 mmol/l (5.4–5.9) pre-pregnancy, 4.8 mmol/l (4.8–4.8) throughout pregnancy and 5.8 mmol/l (5.5–5.8) post-delivery. Insulin action time was 4.0 (4.0–4.0) hours during pregnancy and post-delivery.

The amount of carbohydrate entered in the insulin pumps was stable ~ 145 g during pregnancy and post-delivery.

isCGM metrics (Table 2) were similar between insulin pump and MDI users. Insulin doses are presented in Table 3.

**Table 2:** Intermittently scanned continuous glucose monitoring data seven days prior to randomisation and at 21 weeks and 33 weeks in 117 women with type 1 diabetes using non-automated insulin pumps or multiple daily injections during pregnancy

	Insulin pumps *n* = 26	Multiple daily injections *n* = 91	*P* value
***Randomisation***			
Mean sensor glucose, mmol/l	7.0 (6.3–7.8)	6.6 (6.2–7.5)	0.41
Percentage of time below range (< 3.9 mmol/l)	8.0 (3.0–11.0)	7.0 (2.0–14.0)	0.60
Percentage of time in range (3.9–7.8 mmol/l)	52.3 (± 20.9)^a^	61.7 (± 15.7)^b^	0.06
Percentage of time above range (> 7.8 mmol/l)	42.4 (± 23.3)^a^	29.1 (± 16.0)^b^	0.06
***21 weeks of gestation***			
Mean sensor glucose, mmol/l	6.9 (± 1.0)	6.8 (± 1.0)	0.67
Percentage of time below range (< 3.9 mmol/l)	7.0 (5.5–13.0)	8.0 (4.5–13)	0.91
Percentage of time in range (3.9–7.8 mmol/l)	60.9 (± 16.5)^c^	62.8 (± 14.0)^c^	0.62
Percentage of time above range (> 7.8 mmol/l)	23.0 (21.2–37.2)^c^	27.0 (18.0–39.0)^c^	0.98
***33 weeks of gestation***			
Mean sensor glucose, mmol/l	6.3 (5.8–6.7)	6.3 (5.9–6.6)	0.80
Percentage of time below range (< 3.9 mmol/l)	8.5 (3.5–12.0)	7.0 (3.0–12.0)	0.67
Percentage of time in range (3.9–7.8 mmol/l)	67.5 (60.2–77.2)^d^	72 (65.2–81.0)	0.27
Percentage of time above range (> 7.8 mmol/l)	27.0 (13.5–27.8)^d^	19.0 (11.0–26.8)	0.22

Data are presented as *n* (%), mean (standard deviation) or median (interquartile range)

Data availability at randomisation: >89%, except for ^a^54% and ^b^74%

Data availability at 21 weeks: >88%, except for ^c^>69%

Data availability at 33 weeks: >84%, except for ^d^69%

**Table 3:** Insulin doses before, during and after pregnancy in 141 women with type 1 diabetes using non-automated insulin pumps or multiple daily injections during pregnancy

	Pre-pregnancy	Randomisation	21 weeks	33 weeks	35 weeks	1 month post-delivery	3 months post-delivery
**Insulin pumps (n = 39)**							
Total daily insulin dose							
- *IU/day*	37.0 (30.5–50.3)	38.8 (32.2–51.0)	45.0 (36.2–55.2)	72.4 (57.6–91.8)	78.8 (58.3–102.0)^b^	35.0 (29.0–40.0)	36.0 (32.1–43.3)
- *IU/kg/day*	0.5 (0.5–0.7)	0.5 (0.5–0.6)	0.6 (0.5–0.7)	0.8 (0.7–1.1)	0.9 (0.7–1.1)^c^	0.5 (0.4–0.5)	0.5 (0.4–0.5)^d^
Basal insulin, percentage of total daily insulin dose	50.5 (43.8–56.8)^a^	51.0 (43.0–57.0)	42.0 (36.5–45.0)	36.0 (33.0–41.9)	37.0 (32.0–42.5)^c^	48.0 (44.0–53.5)	47.0 (40.0–52.0)
**Multiple daily injections (n = 102)**							
Total daily insulin dose							
- *IU/day*	39.0 (28.6–48.0)	33.0 (26.0–45.6)	40.0 (29.0–51.8)	68.2 (49.8–88.2)	61.5 (50.2–88.0)	30.0 (23.0–40.0)	35.0 (25.0–47.1)
- *IU/kg/day*	0.5 (0.4–0.7)	0.5 (0.4–0.6)	0.5 (0.4–0.7)	0.9 (0.6–1.0)	0.7 (0.6–1.0)	0.4 (0.3–0.5)	0.5 (0.4–0.6)
Basal insulin, percentage of total daily insulin dose	56.3 (48.8–63.3)	52.3 (45.6–57.5)	44.6 (36.6–53.6)	40.0 (31.6–49.5)	38.0 (31.9–45.4)	50.0 (39.3–58.8)	48.8 (40.0–54.5)

Data are presented as median (interquartile range)

Data availability for insulin pumps: Pre-pregnancy data > 87%, except for ^a^72%. Pregnancy data ≥ 95%, except for ^b^87% and ^c^85%. Post-delivery data > 77%

Data availability for multiple daily injections: Pre-pregnancy data > 95%. Pregnancy data > 87%. Post-delivery data > 74%

The prevalence of preterm delivery was 25.9% vs. 16.7% (*P* = 0.01). In total, 64 (45%) women delivered by caesarean section, corresponding to 59.0% in the insulin pump users vs. 40.2% in the MDI users (*P* = 0.04) (Table 4).

**Table 4:** Maternal, pregnancy, neonatal and fetal outcomes in 141 women with type 1 diabetes using non-automated insulin pumps or multiple daily injections during pregnancy

	Insulin pumps *n* = 39	Multiple daily injections *n* = 102	*P* value
**Maternal and pregnancy outcomes**			
HbA_1c_ at 35 weeks, mmol/mol^a^	43 (40–46)	43 (39–46)	0.53
HbA_1c_ at 35 weeks, %^a^	6.1 (5.8–6.4)	6.1 (5.7–6.4)	0.53
Gestational weight gain, kg^b^	15.5 (10.1–16.9)	13.8 (10.9–17.6)	0.71
Number of participants with excessive gestational weight gain^c^	14 (35.9)	30 (29.4)	0.48
Preeclampsia	7 (17.9)	10 (9.8)	0.25
Gestational age at delivery, days	259 (± 9.2)	264 (± 8.1)	0.001
Vaginal delivery, total	16 (41.0)	61 (59.8)	0.04
- *Non-instrumental*,* vaginal delivery*	*13*	*54*	
- *Instrumental*,* vaginal delivery*	*3*	*7*	
Caesarean section, total	23 (59.0)	41 (40.2)	
- *Planned caesarean section*	*14*	*24*	
- *Emergency caesarean section*	*9*	*17*	
Early preterm delivery (< 34 weeks)	1 (2.6)	1 (1.0)	0.48
Preterm delivery (< 37 weeks)	14 (35.9)	17 (16.7)	0.01
Number of participants with severe hypoglycaemic events during pregnancy, *from randomisation until delivery*	2 (5.1)	5 (4.9)	1.0
**Neonatal outcomes**			
Birthweight standard deviation score	1.51 (± 1.21)	1.21 (± 1.34)	0.23
Birthweight, g	3530 (± 453)	3592 (± 536)	0.52
Length, cm	51 (50–52)	52 (50–53)	0.54
Head circumference, cm	35 (34–36)	35 (34–36)	0.23
Abdominal circumference, cm^d^	34 (31.8–35)	34 (32–35)	0.71
Large for gestational age	25 (64.1)	51 (50.0)	0.13
Small for gestational age	0 (0.0)	2 (2.0)	1.0
Neonatal hypoglycaemia, plasma glucose < 2.2 mmol/l during first two hours after birth	6 (15.4)	12 (12.0)	0.59
Neonatal jaundice requiring photo therapy^e^	8 (24.2)	18 (19.6)	0.57
Respiratory distress requiring Continuous Positive Airway Pressure treatment	10 (25.6)	21 (20.6)	0.52
Neonatal morbidity (neonatal hypoglycaemia, respiratory distress, and/or jaundice requiring photo therapy)	21 (53.8)	39 (39.0)	0.11
Admission to Neonatal Intensive Care Unit	14 (35.9)	23 (22.5)	0.11
Duration of stay at Neonatal Intensive Care Unit, days	2.5 (1–3)	2 (1–3.5)	0.88
Admission to Neonatal Intensive Care Unit > 48 h	7 (17.9)	10 (9.8)	0.25
**1 month post-delivery**			
Maternal HbA_1c_, mmol/mol^f^	43 (41–48)	43 (41–48)	1.0
Maternal HbA_1c_, %^f^	6.1 (5.9–6.5)	6.1 (5.9–6.5)	1.0
Breastfeeding, any^g^	21 (84.0)	48 (82.7)	1.0
Number of mild hypoglycaemic events the previous week	4 (2–7)	3 (2–7)	0.31
Infant weight standard deviation score	– 0.09 (– 0.7–0.6)	– 0.09 (– 0.75–0.6)	0.96
Infant weight, g	4280 (± 653)	4339 (± 572)	0.61
Infant length, cm^h^	53.8 (± 2.5)	54.3 (± 2.5)	0.35
Number of infants hospitalised since discharge after delivery	8 (21.6)	10 (11.6)	0.15
Number of days with infant hospitalisation since discharge after delivery	3 (2–5.5)	3 (1.25–4)	0.62
**3 months post-delivery**			
Maternal HbA_1c_, mmol/mol^i^	52 (48–57)	49 (45–56)	0.14
Maternal HbA_1c_, %^i^	6.9 (6.5–7.4)	6.6 (6.3–7.3)	0.14
Breastfeeding, any^j^	18 (75.0)	52 (78.8)	0.78
Number of mild hypoglycaemic events the previous week	2 (1–5)	2.5 (1.6–5)	0.94
Number of participants with severe hypoglycaemic events since delivery	1 (2.6)	4 (3.9)	1.0
Maternal post-delivery weight retention, kg^k^	2.7 (± 5.3)	3.6 (± 5.4)	0.44
Number of participants with post-delivery weight retention > 5.0 kg compared to pre-pregnancy weight^k^	8 (25.0)	28 (36.8)	0.23
Infant weight standard deviation score	– 0.25 (– 0.95–0.75)	– 0.19 (– 0.64–0.39)	0.80
Infant weight, g	5900 (5345–6850)	6020 (5600–6500)	0.66
Infant length, cm^l^	61 (59.8–62.1)	61 (58.8–62)	0.74
Number of infants hospitalised since last trial visit	2 (6.1)	3 (3.6)	0.62
Number of days with infant hospitalisation since last trial visit	2.5 (1.75–3.25)	2 (1.5–4)	1.0

Data are presented as mean (standard deviation), median (interquartile range) or n (%)

Data availability for maternal and pregnancy outcomes: >99%, except for ^a^89.7% and 85.3%, and ^b^87.2% and 86.3%

^c^Excessive gestational weight gain is defined as gestational weight gain exceeding the recommendation by National Academy of Medicine: pre-pregnancy BMI < 25 kg/m2: ≥ 16 kg, pre-pregnancy BMI 25–29.9 kg/m2: ≥ 11.5 kg and pre-pregnancy BMI of ≥ 30 kg/m2: ≥ 9.0 kg

Data availability for neonatal outcomes: >95%, except for ^d^82.1% and 91.2%, and ^e^84.6% and 90.2%

Data availability for 1 month post-delivery: >84%, except for ^f^71.8% and 51.0%, ^g^64.1% and 56.9% and ^h^79.4% in the multiple daily injection group

Data availability for 3 month post-delivery: >79% except for ^i^59.0% and 46.1%, ^j^61.5% and 64.7%, ^k^74.5% in the multiple daily injection group and ^l^71.8% and 69.6%

Univariate logistic regression analysis identified insulin pump use (OR 2.8 (95% CI 1.2–6.5, *p* = 0.02)) and microvascular complications (OR 2.3 (95% CI 0.99–5.7, *p* = 0.054)) as possible risk factors for preterm delivery. In multivariate logistic regression analysis insulin pump use remained a risk factor for preterm delivery (OR 3.4 (95% CI 1.3–8.5, *p* = 0.01) independent of other possible risk factors.

Univariate logistic regression analysis identified insulin pump use (OR 2.1 (95% CI 1.02–4.6, *p* = 0.047)) and microvascular complications (OR 2.0 (95% CI 0.96–4.2, *p* = 0.07)) as risk factors for caesarean section. In multivariate logistic regression analysis insulin pump use remained a risk factor for caesarean section (OR 2.6 (95% CI 1.1–6.2, *p* = 0.02)) independent of other possible risk factors.

## Discussion

This preplanned secondary analysis of the CopenFast trial included 141 pregnant women with type 1 diabetes stratified by insulin treatment modality (insulin pump or MDI) who used CGM routinely. Despite comparable isCGM metrics and HbA_1c_, insulin pump users had higher prevalence of preterm delivery and caesarean sections, compared to MDI users, which was independent of diabetes duration, age and presence of microvascular complications. In insulin pump users at 33 weeks, basal insulin rates were ~ 37% higher and carbohydrate-to-insulin ratio ~ 61% lower compared to pre-pregnancy, and at 3 months post-delivery basal insulin rates were ~ 20% lower and carbohydrate-to-insulin ratios were similar to pre-pregnancy.

All insulin pump users were advised to adjust insulin pump settings at the routine visits and every 3–5 days between visits, as indicated, and they were mainly treated by the same two diabetologists. The changes in insulin pump settings during pregnancy and post-delivery were overall consistent with previous small studies at our centre [10, 11]. Insulin doses, in particular mealtime insulin doses, increased from randomisation and throughout pregnancy in agreement with what is expected in pregnant women with type 1 diabetes [14, 15, 16].

Overall, 45% of the women delivered by caesarean section, consistent with previous data from our centre between 2012 and 2016 [4]. However, insulin pump users delivered significantly more often preterm and by caesarean sections, compared to MDI users.

It is reassuring that the rate of breastfeeding was high and no differences in infant health were detected between the two groups. Given the relatively low number of women on insulin pumps in this secondary analysis, we did not exclude the few women who did not breastfeed, when evaluating insulin pump settings post-delivery.

Our findings of insulin pump users not achieving improved pregnancy outcomes compared to MDI users may be ascribed confounding by indication as the insulin pump users were significantly younger at diabetes diagnosis and at baseline. Almost half of all insulin pump users had microvascular complications which is in line with childhood-onset type 1 diabetes being associated with more frequent development of microvascular complications [34, 35].

It is natural to consider that the non-automated insulin pumps in this analysis lacked the ability to achieve tight glycaemic control, and the precision needed to accommodate the changing insulin requirements during pregnancy [10]. Recently, automated insulin delivery (AID) systems are available, integrating insulin pumps with CGMs to automatically detect changes in blood glucose levels and, based on algorithms integrated in mobile applications, to adjust the insulin doses accordingly. RCTs have examined the effect of AID systems in women with type 1 diabetes [36, 37]. None of these studies were powered for pregnancy outcomes and studies on the effect of AID on pregnancy outcomes are warranted.

Mean sensor glucose was ~ 7.0 mmol/l at randomisation and 6.3 mmol/l at 33 weeks with use of isCGM, and overall targets for glycaemic metrics were not achieved during pregnancy. In the CopenFast trial CGM data were only available for women using isCGM and were taken from patient records seven days prior to randomisation and at 21 and 33 weeks, whereas data on rtCGM were not collected continuously during pregnancy in the small subset of women using this device. Continuous use of rtCGM data from peri-conception until delivery, if available, might have unmasked differences in glycaemic metrics which were not detectable with isCGM in the present analysis.

Limitations include that the open-label design may have introduced bias, due to the influence on the investigators’ and the women’s expectations to the different treatments. Data on indications for planned caesarean section or, in multiparous women, previous caesarean section were not collected which precludes comparisons between groups. The amount of carbohydrate entered in the insulin pumps was stable during pregnancy and post-delivery. However, it is not known whether the women entered all carbohydrates consumed, and they may have refrained from entering carbohydrates consumed for hypoglycaemia treatment. Data on diet and physical activity were not registered, but gestational weight gain was similar in the two groups. Adjustment for multiple testing was not performed therefore a type 1 error cannot be ruled out. Data on insulin pump settings and CGM metrics imply a high number of comparisons given the nature of these data collected and compared across pregnancy. This preplanned secondary analysis presented secondary outcomes of the CopenFast trial as predefined in the published protocol [24]. However, interpretation of the findings should be made with the reservation that correction for multiple testing was not performed.

Strengths of this preplanned secondary analysis include that it was based on an RCT where women were stratified by usual insulin treatment modality with insulin pump or MDI. The trial medication was equally distributed in both groups and therefore most likely did not influence the outcomes of the present analysis. Data were collected prospectively, and all women came from the same well-defined geographical area and attended the same specialised diabetes unit. This ensured that all women were treated according to the same standardised treatment guidelines, however, this could possibly also reduce the generalisability of the results. Approximately 90% of the women were of Nordic origin, and only a few exclusion criteria were applied, which enhanced the generalisability of the findings to the Nordic female population, although it might reduce the applicability to other ethnic groups. Data availability was high, with > 95% availability for most variables during pregnancy. Post-delivery data availability was ~ 80% for most outcomes, which we consider impressive as the cohort consisted of women who, besides having to take care of the newborn, also had to deal with their chronic condition type 1 diabetes on a daily basis.

The randomised and stratified design of the original trial offers a certain robustness of the findings of this secondary analysis. However, multiple factors affect pregnancy outcomes in women with type 1 diabetes. This, together with the relatively low number of women in this analysis, calls for caution when interpreting the results.

In conclusion, women with type 1 diabetes using non-automated insulin pumps did not achieve improved pregnancy outcomes compared to women using MDI. This was despite all using CGM continuously throughout pregnancy and achieving comparable isCGM metrics and HbA_1c_ values. However, the secondary observational design limits the ability to make causal inferences. Future studies on new diabetes technology with AID and rtCGM are needed to evaluate the impact on pregnancy outcomes.
