# Correction: Treatment with non-automated insulin pumps or multiple daily injections during pregnancy and post-delivery in women with type 1 diabetes: A secondary analysis of the copenfast trial

**DOI:** 10.1007/s00592-025-02593-1

**Published:** 2025-12-05

**Authors:** Katrine Christiansen, Sidse K. Nørgaard, Kirsten Nørgaard, Tine D. Clausen, Peter Damm, Elisabeth R. Mathiesen, Lene Ringholm

**Affiliations:** 1 Department of Nephrology and Endocrinology, Rigshospitalet, Copenhagen, Denmark; 2 Department of Clinical Medicine, Faculty of Health and Medical Sciences, University of Copenhagen, Copenhagen, Denmark; 3 Center for Pregnant Women with Diabetes, Rigshospitalet, University of Copenhagen, Blegdamsvej 9, Copenhagen, 2100 Denmark; 4 Novo Nordisk, Søborg, Denmark; 5 Steno Diabetes Center Copenhagen, Herlev, Denmark; 6 Department of Gynaecology, Fertility, and Obstetrics, Rigshospitalet, Copenhagen, Denmark

**Correction: Acta Diabetologica**

10.1007/s00592-025-02560-w

In the original version of this article, the affiliation details were incorrectly given as “Center for Pregnant Women with Diabetes, Department of Nephrology and Endocrinology, Rigshospitalet, University of Copenhagen, Blegdamsvej 9, Copenhagen 2100, Denmark” but should have been “Center for Pregnant Women with Diabetes, Rigshospitalet, University of Copenhagen, Blegdamsvej 9, Copenhagen 2100, Denmark”. And also Table 1, 3 and 4 were incorrectly structured. The original article has been corrected.

